# Case Report: Paternal Uniparental Isodisomy and Heterodisomy of Chromosome 16 With a Normal Phenotype

**DOI:** 10.3389/fped.2021.732645

**Published:** 2021-10-22

**Authors:** Xu Zhang, Li Liu, Yang Liu, Xin Pan

**Affiliations:** The Department of Obstetrics and Gynecology, The Second Affiliated Hospital of Chongqing Medical University, Chongqing, China

**Keywords:** paternal uniparental disomy, UPD 16, SNP array, prenatal diagnosis, chromosomal variant

## Abstract

Uniparental disomy (UPD) is a specific type of chromosomal variant that has been detected in both prenatal diagnosis and neonates with advances in molecular genetic testing technologies [mainly chromosome microarray analysis (CMA) technologies containing single-nucleotide polymorphism (SNP) probes]. In this case, we performed non-invasive prenatal genetic testing (NIPT) to screen fetuses for aneuploidy and detected the presence of aneuploidy chimerism and UPD by CMA, including SNP analysis and whole-exome sequencing, to detect pathogenic variants within the genome. The NIPT results suggested an increased number of fetal chromosome 16, and the CMA results indicated that it was the first case of holistic paternal UPD16 with isodisomy combined with heterodisomy, although no abnormal phenotype was seen in the newborn at postnatal follow-up. The homozygous region of the isodimer combined with the heterodimer is smaller than that of the complete isodimer, and it is less prone to recessive genetic diseases. A retrospective analysis of this case of paternally derived UPD16 was used to explore the uniparental diploid origin of chromosome 16 and to provide some reference for genetic counseling and prenatal diagnosis.

## Introduction

Eric Engel first proposed the concept of a single-parent diploid in 1980, which described the situation in which two homologous chromosomes are inherited from the same parent and have no genetic relationship with the other parent (1). The incidence of uniparental diploidy in newborns is ~0.029%. As of 2010, statistics in the literature have reported ~1,100 cases of whole uniparental disomy (UPD) and 120 cases of partial UPD (2). Recent data from more than 4 million subjects studied by the personal genetics companies 23 and Me and Biobankhave led to the estimation that all chromosomes (not only chromosomes with imprinted regions) have a UPD incidence of 1/2,000 (3). Chromosome 16 is one of the chromosomes that are prone to non-integration, which also causes a high incidence of chromosome 16 UPD, but most cases are maternal UPD. According to statistics from Kotzor and other scholars in 2005, there have been more than 50 cases of maternal chromosome 16 UPD reported in the literature, and their clinical phenotypes have varied, ranging from no abnormal clinical phenotypes to mental retardation, developmental delay, and structural abnormalities (4). However, only two cases of paternal UPD16 have been reported, and its clinical manifestations range from no abnormal clinical manifestations to the onset of Mendelian genetic disease (5, 6).

UPD includes three subtypes: heterodisomy, isodisomy, and partial isodisomy. Heterodisomy is caused by non-segregation in stage I meiosis, and the affected individual inherits two homologous chromosomes from the same parent; isodisomy is caused by non-segregation in stage II meiosis, and the affected individual inherits two sister chromatids of one homologous chromosome from one parent. For the simple type of uniparental diploid (referring to the absence of combined trisomy or other abnormal mosaicism), its pathogenicity mainly lies in two aspects. One is caused by the influence of imprinted genes from this perspective. In other words, most chromosomal UPDs do not have clear and characteristic clinical symptoms. Currently, UPDs on chromosomes 6, 7, 11, 14, 15, and 20 can cause clinical symptoms. The second type is the onset of recessive genetic diseases on the chromosome where the UPD is located. For example, UPD on the X chromosome may cause the onset of X-linked recessive genetic diseases in female patients (7). As the known cases of UPD on chromosome 16 belong to the overall UPD involving the entire chromosome, the segments and genes that cause UPD on chromosome 16 cannot be located, and its pathogenic mechanism is also difficult to analyze. Although partial UPD16 cases involving only partial fragments of chromosome 16 can help researchers determine the chromosome segment that causes specific clinical symptoms, further determine the key genes that cause the disease, and clarify gene functions and pathogenic mechanisms, there is no literature support at present.

This study used single-nucleotide polymorphism (SNP) array technology to perform copy number analysis and SNP genome typing on a prenatal diagnosis sample with abnormal non-invasive prenatal genetic testing (NIPT) and found a case of paternal UPD16. The results of the whole-exome sequencing (WES) test showed no abnormalities, the neonatal follow-up after birth did not show abnormal phenotypes, and all developmental indicators were normal. Similar to the previous two reports on paternal upd16, no abnormal syndrome was found in the cases. The cases reported by Kohlhase et al. found bilateral calcaneal and mandibular arch hypoplasia. In the cases reported by Donova et al., Fanconi anemia was mainly caused by homozygous mutation of the *FANCA* gene (5, 6). No significant gene mutation on the chromosome of UPD was found in this case report; the two previous reports were isodimers, whereas this case report describes isodimers combined with heterodimers. Compared with a complete isodimer, the homozygous region is relatively small, and the risk of recessive diseases is lower. This study conducted a retrospective analysis of this case of UPD16 to explore the source and pathogenic mechanism of chromosome 16-UPD and its application value in clinical response and genetic counseling.

## Clinical Report

**Figure 1:**
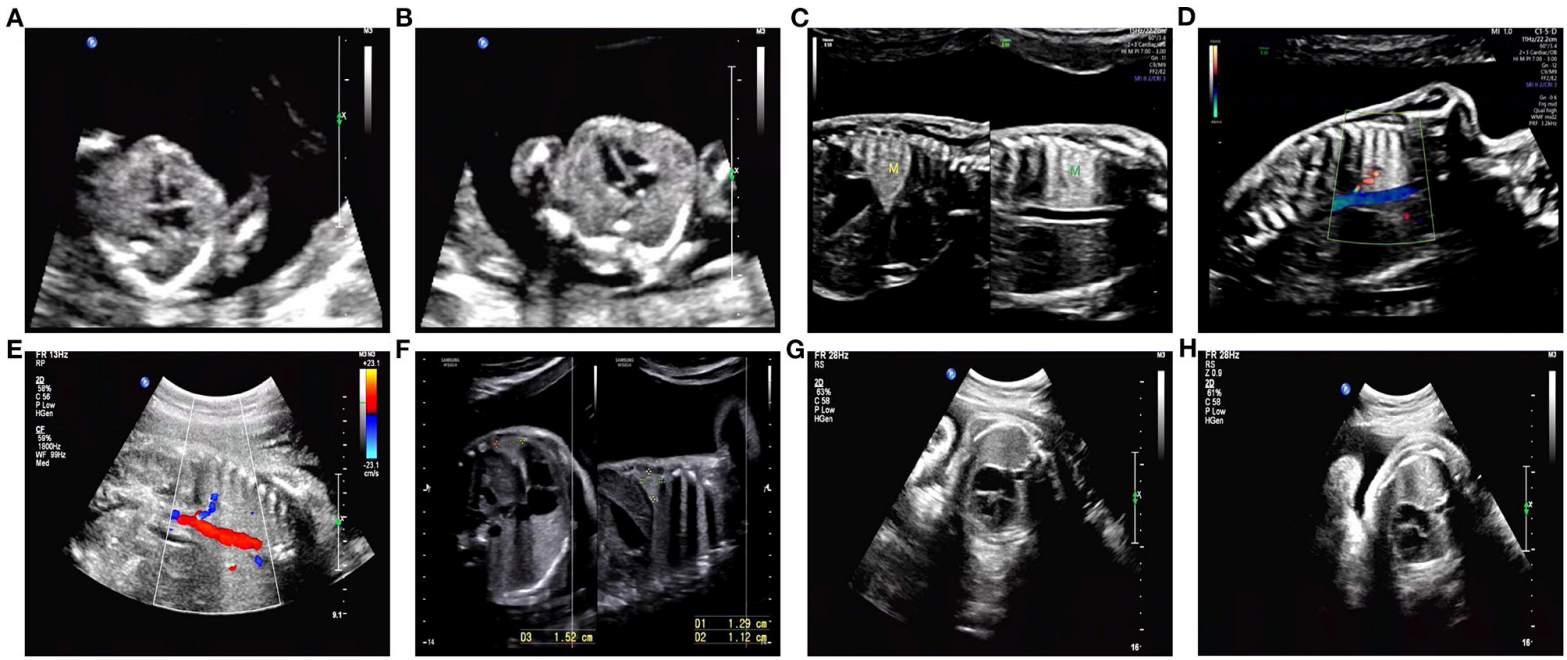
Detailed ultrasound images. **(A,B)** No abnormalities were observed in this fetus at 12 weeks of gestation. **(C,D)** An ultrasound scan revealed abnormal echogenicity of the left lower lung (isolated lung possible) and hydramnion at 25 weeks of gestation. **(E,F)** Ultrasound image of abnormal echogenicity of the left lower lung (isolated lung possible) and normal amniotic fluid volume at 30 weeks of gestation. **(G,H)** At 34 and 37 weeks of gestation, no abnormalities were observed on ultrasound.

We present a case of a 26-year-old pregnant woman, G2P0 (gravida 2, para 0), with both pregnancies from the same non-consanguineous male partner. The couple had normal physical conditions, normal mental development, and no adverse contact or exposure in the working environment. The first abortion of the pregnant woman was an induced abortion, and the aborted tissue was not examined for genetics. The present pregnancy was spontaneous. The results of NIPT at 13 weeks of gestation showed that the number of chromosomes at 16 was excessive. Chromosome microarray analysis (CMA) results after amniocentesis at 18 weeks showed that there were two regional homozygous fragments around the centromere of chromosome 16. CMA family analysis suggested that fetal chromosome 16 was an integral paternal UPD with isodisomy and heterodisomy. Consecutive systematic ultrasound examinations throughout pregnancy were as follows: ultrasonography at 12 weeks of gestation showed no significant abnormality (Figures 1A,B). On ultrasound performed at 25 weeks, abnormal echogenicity of the fetal left lower lung (possible isolated lung) and slight polyhydramnios were observed (Figures 1C,D). At 30 weeks of gestation, the ultrasound results were the same as before, but the amniotic fluid volume had returned to normal (Figures 1E,F). Ultrasonography at 34 and 37 weeks showed no significant abnormality (Figures 1G,H). There were no abnormalities in blood pressure, weight, uterine height, or abdominal circumference during pregnancy and no abnormalities in any fetal heart rate test. In the first trimester of pregnancy, she had been treated with infusion for cold and fever, but the specific medication was unknown. The pregnant woman delivered a boy by cesarean section at 38 weeks 3 days. A physical examination was performed after birth, and the weight of the newborn was 3.25 kg.

At 1 month and 5 days old, the baby's weight was 4.7 kg, his height was 52.0 cm, his head circumference was 39.0 cm, his facial features were normal, his mental development was within the normal range, his prehalogen was 2.0 × 2.0 cm, and his hearing oral, chest, abdominal, and umbilical examinations were unremarkable. At 4 months 18 days old, the baby's weight was 6.8 kg, his height was 59.0 cm, his head circumference was 43.5 cm, his facial features were normal, his mental condition was good, his front halogen was 1.0 × 1.0 cm, his physical examination is normal, and his movement and language development were normal. At 6 months old, the baby's weight was 7.2 kg, his height was 64.0 cm, his head circumference was 43.8 cm, his front halogen was 1.0 × 1.0 cm, his physical examination showed no abnormalities, and his hemoglobin value was 105 g/L. At 10 months 26 days old, the baby's weight was 8.9 kg, his height was 70.5 cm, and his head circumference was 48.9 cm. There were no abnormalities in facial features or in his gross motor, fine motor, or speech development. After birth, peripheral blood was retrieved for karyotyping and SNP array analysis, and the results were consistent with the prenatal results. A WES test was also performed and showed no abnormal results. Overall, to the date of this article, the clinical presentation of the newborn did not show any adverse conditions. All data were collected after obtaining informed consent from the patient.

## Materials and Methods

### Non-invasive Prenatal Genetic Testing

Peripheral venous blood (5 mL) was collected from the pregnancy at 19 weeks of gestation, anticoagulated by ethylenediaminetetraacetic acid, and then transferred into a sterile centrifuge tube for NIPT (The Beijing Genomics Institute). The procedure was performed as described in a previous study (8). Then, cell-free DNA from plasma was extracted and stored at −80°C. The kits were purchased from BGI Biotechnology Co., Ltd. (Wuhan, China). Cell-free DNA was sequenced using the MGISeq-2000 sequencing system (BGI, China) to obtain the exact DNA fragment distribution on each chromosome. The coverage depths (Cov-chrN) of the test and standard samples were calculated based on bioinformatics analysis and then converted into a specific risk index, which was eventually used to determine the sample risks of trisomy 21 (T21), trisomy 18 (T18), and trisomy 13 (T13).

### Cytogenetic Analysis

Peripheral blood samples were collected from both parents. Chromosome analysis was performed according to the standard protocol using G-banding at a 450-band resolution. At least 25 metaphases were read for each sample.

### Chromosome Microarray Analysis

In this study, an SNP array was used to confirm the existence of genomic variation that was detected by NIPT. Genomic DNA was extracted from peripheral blood or amniotic fluid cells from pregnant women using the QIAamp DNA Mini Kit (Qiagen, Hilden, Germany). The Infinium Global Screening Array (Illumina, San Diego, CA), consisting of ~700,000 marker genome-wide tag SNPs and markers targeting all regions of known cytogenetic importance, was applied for the whole-genome scan. Molecular karyotype analysis was performed using GenomeStudio V2011.1 software (Illumina, San Diego, CA). Automated detection of copy number changes was carried out using the cnvPartition algorithm (versions 1.2.1–3.1.6) in GenomeStudio V2011.1 software. All identified abnormalities were further characterized by visual inspection of the Log R and BAF chromosomal plots (9).

### WES Analysis

WES is a high-throughput sequencing analysis that captures and enriches DNA from all exome regions of the genome using exome sequence-specific capture technology. WES analysis was performed by BGI-Shenzhen Clinical Laboratory Centre. The exome describes the protein-coding regions of the human genome; most pathogenic gene mutations occur in exome regions. WES captures probes that cover only 1–1.5% of the human genome, allowing for the accurate detection of multiple exome disease-causing variants at once (10). Genomic DNA from the blood of the subject was used as the test material. The DNA was first sheared, and libraries were prepared. Then, the exons of the target gene and the DNA in the adjacent shear region were captured and enriched by the BGI V4 chip. Finally, the MGISEQ-2000 sequencing platform (Shenzhen, China) was used for variant detection. Sequencing data quality control indicators were as follows: the average sequencing depth of the target region was ≥ 180 ×, and the percentage of loci with average depth >20 × in the target region was >95%.

### Data Analysis

Sequenced fragments were aligned to the UCSC hg19 human reference genome by BWA to remove duplicates. GATK was used for base mass value correction and SNV, INDEL, and genotype detection. Exome depth was used for copy number variation detection at the exon level. The specific experimental procedure was performed according to the kit instructions (11–15).

We evaluated the chromosome region with the information provided by the Online Mendelian Inheritance in Man database (OMIM, http://omim.org/), the DECIPHER Database (http://decipher.sanger.ac.uk), the UCSC database (http://genome.ucsc.edu), and the Geneimprint database (http://www.geneimprint.com/).

## Results

### Results of NIPT

The NIPT of this case report indicated that the fetus had a high risk of an increased number of chromosome 16 and no other chromosomal abnormalities (data not shown). Further amniocentesis or cord blood aspiration for karyotyping and gene chip analysis was required to confirm the diagnosis.

### Karyotype Results

**Figure 2:**
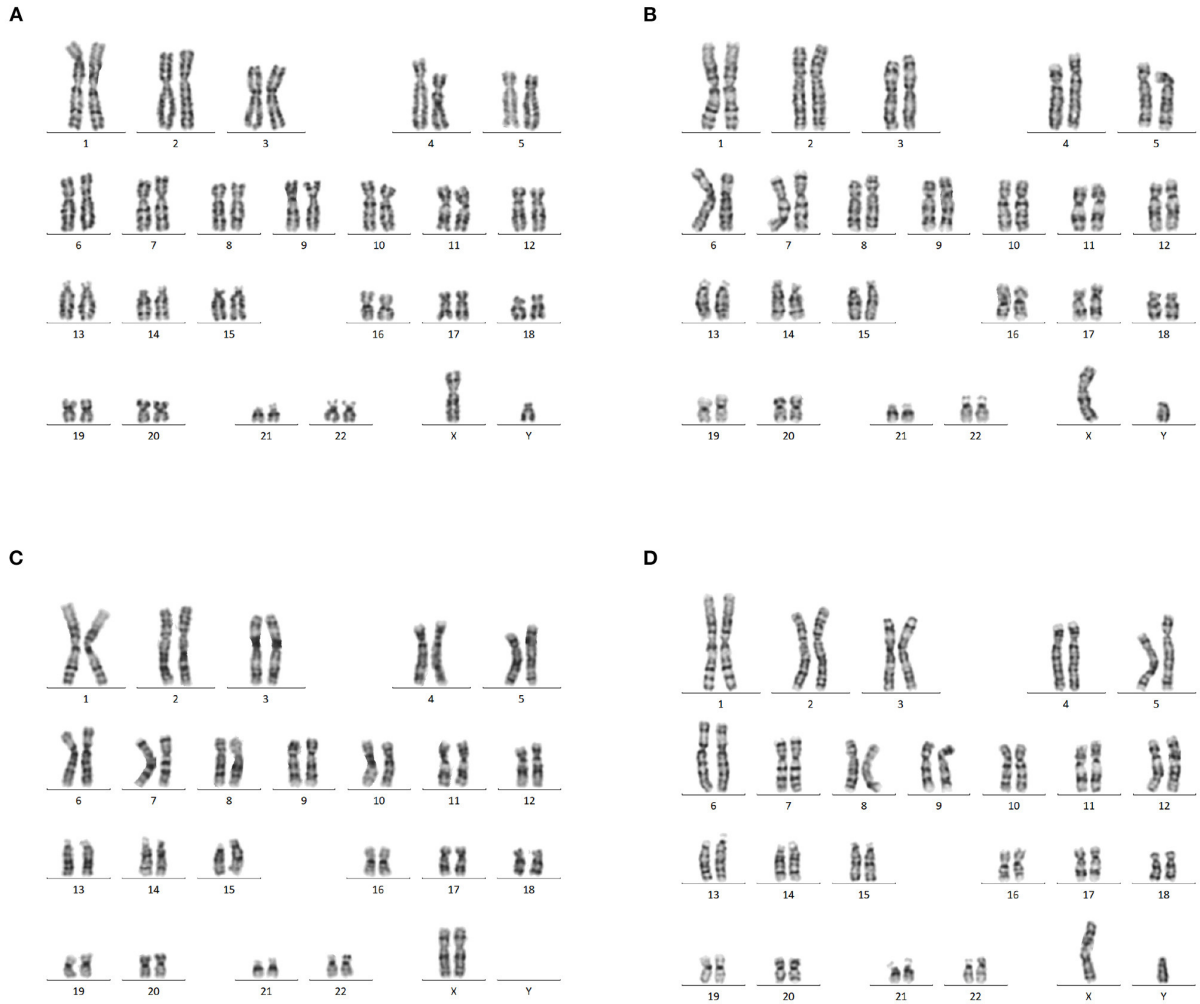
Karyotype analysis. **(A)** Normal karyotype analysis of amniotic fluid cells (46,XY). **(B)** Karyotype analysis of newborn peripheral blood, showing a 46, XY karyotype as the fetal amniotic fluid sample. **(C,D)** The peripheral blood of the couple showed a normal 46, XX and 46, XY karyotype.

Karyotyping of the fetus, consistent with his newborn's peripheral blood, revealed a normal karyotype (46, XY) (Figures 2A,B). The couple had normal karyotype results (46, XX and 46, XY) (Figures 2C,D).

### CMA Detected UPD of Chromosome 16

**Figure 3:**
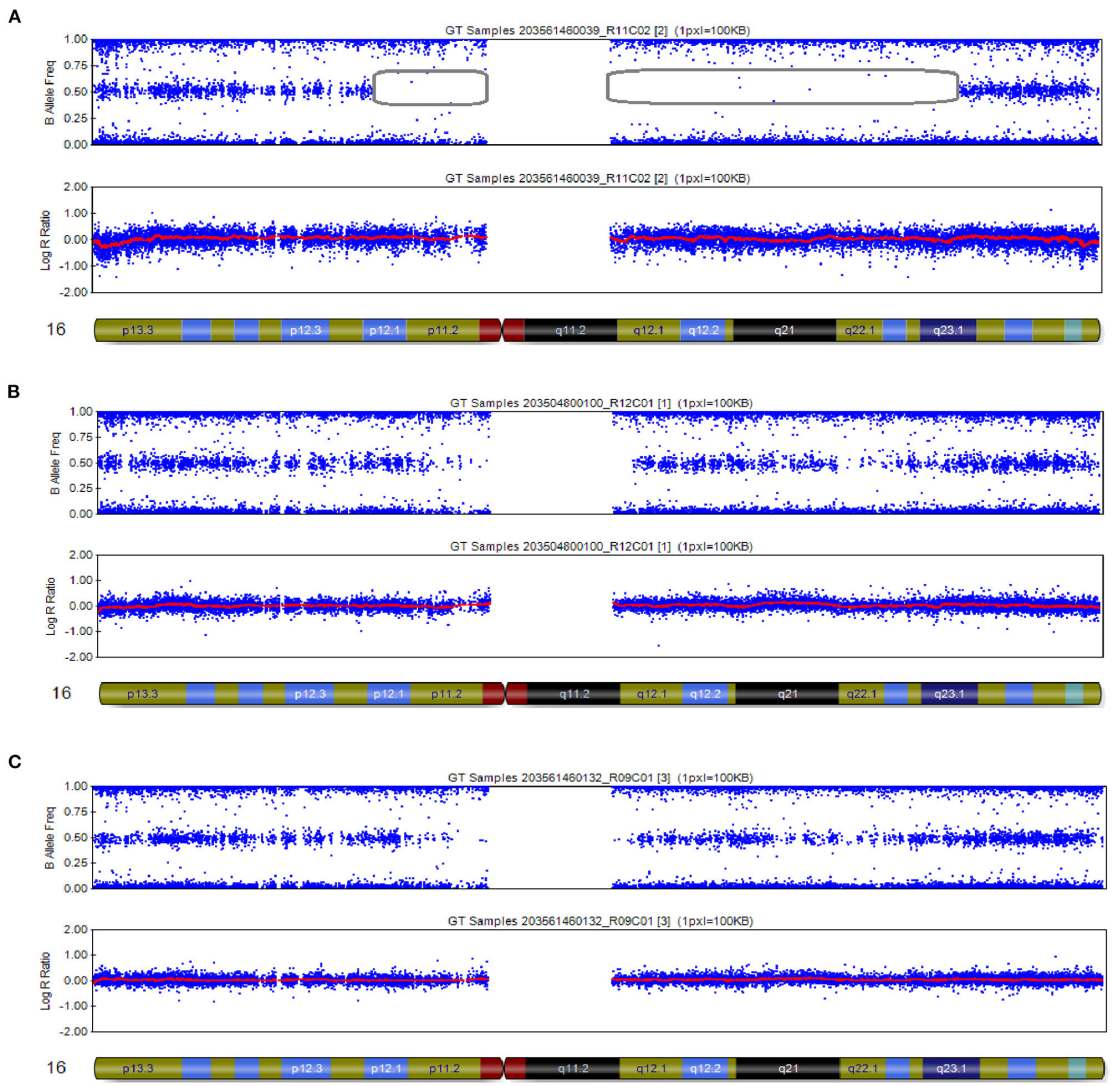
SNP array results of these cases involved imprinted chromosome 16. **(A)** The fetus was chromosome 16 combined with UPD: 16p12.1p11.1 (25,079,459–35,257,261) × 2 hmz pat, 16q11.2q23.1 (46,394,361–77,737,858) × 2 hmz pat. **(B,C)** SNP arrays revealed no abnormalities at chromosome 16 in females and males.

The CMA results of the peripheral blood of the newborn after birth were consistent with the CMA results of the amniotic fluid (16). In this case, the log R ratio of chromosome 16 was consistent with a normal copy number; in addition, some genotypes present were homozygous (genotypes as AA or BB). There was partial isodisomy of chromosome 16 with loss of heterozygosity (genotypes as AB). This is consistent with the mechanism of trisomy/monosomy rescue. Whole-genome SNP array analysis can detect all chromosome number abnormalities; identify and detect chromosome rearrangements, including genomic sequence gains and losses; and are effective in detecting genomic imbalances. In this case, whole-genome SNP array analysis on uncultured amniocytes detected arr [hg19] 16p12.1p11.1 (25,079,459–35,257,261) × 2 hmz and 16q11.2q23.1 (46,394,361–77,737,858) × 2 hmz, which indicated a case of isodimeric merged heterodimeric holomeric paternal UPD (Figure 3A). The results of SNP typing for all chromosomes except chromosome 16 supported the parentage of the fetus to both spouses (Figures 3B,C). The comparative results of typing in neonates are shown in Table 1.

**Table 1:** Comparative results of SNP in neonates.

**Chromosomal segment**	**Length (bp)**	**Total number of SNP probes**	**Number of SNP probe detections**	**SNP probe detection rate**	**Number of AB heterozygotes**	**AB heterozygous ratio**	**Fractal and paternal concordance rate**	**UPD type**
16:88,366–25,068,754	24,980,388	7,419	6,843	92.24%	1,126	16.45%	100%	Heterodisomy
16:25,079,459–35,257,261	10,177,802	1,628	1,490	91.52%	0	0	100%	Isodisomy
16:46,394,361–77,737,858	31,343,497	7,354	6,866	93.36%	0	0	100%	Isodisomy
16:77,741,596–90,161,959	12,420,363	5,273	4,961	94.08%	912	18%	100%	Heterodisomy

### Analysis of Pathogenic Variants in the Genome

In this case, WES did not detect pathogenic/suspected pathogenic variants within the subject's genome.

## Discussion

This case of prenatal diagnosis was clinically specific, with limited testing and difficulty in obtaining comprehensive phenotypic information. The detection and diagnosis method of UPD is based on the relevant guidelines published by the American College of Medical Genetics and Genomics in 2020, which describes the adaptation of UPD detection in prenatal diagnosis. Based on the case we reported, the chromosomal abnormalities detected by NIPT during pregnancy may indicate the existence of UPD. When specific chromosomes are involved, such as chromosomes 6, 7, 11, 14, 15, and 20, we recommend the detection of UPD.

UPD can occur during meiosis of gametes or mitosis of oosperm and is most commonly seen in the q11.2-q13.1 imprinted region of chromosome 15, as in Prader Willi/Angelman. Most of the reported UPDs on chromosome 16 are of maternal origin; as of 2005, more than 50 cases of maternal UPD (16) have been reported, whereas only two cases of paternal UPD have been reported (Table 2).

**Table 2:** Case review of the f parentage UPD16.

**Date**	**Author**	**UPD detection method**	**Isodimer/heterodimer**	**Genetic mutation**	**Sex**	**Age**	**Phenotype**
2000	Kohlhase et al. (5)	STR	Isodimers (technical limitation, cannot confirm the presence of heterodimeric regions)	Untested	Female	Prenatal—13 months	Normal phenotype and no syndromic picture with the exception of bilateral achilles and mandibular arch hypoplasia
2016	Donovan et al. (6)	STR, SNP array	Complete isomorphism	FANCA homozygous mutation (inherited from father)	Female	9 years old	No synthetic picture with the exception of Fanconi anemia that due to the homozygous state of FANCA gene.

Chromosome 16 in this case was identified as a chromosomal paternal UPD with an isodimeric merger of heterodimers based on SNP typing results. Thus, our report is the first confirmed case of a parental UPD (16) with both regional isodimers and regional heterodimers. This case is a newborn boy currently without any abnormal phenotype. The mechanisms of occurrence of complete and regional isodysomy are different. Chromosomal errors occur at different stages of cell division, and types of UPDs may not have the same effects on fetal development.

UPD is usually caused by two non-disjunction events, the first occurring during meiosis and the second during mitosis. Meiosis I non-disjunction is the failure of two homologous chromosomes to separate, resulting in an increased probability of two different homologous chromosomes or uniparental heterodimers from the same parent. After fertilization with a normal haploid gamete, the chromosomes affected in the zygote may be trisomic or monosomic. Mitotic non-disjunction after the formation of a zygote may then occur as a second event, with aneuploidy being rescued by the loss of a third chromosome (trisomic rescue) or the duplication of a monosomic chromosome (monosomic rescue) (7). Given that most non-disjunctions occur in maternal meiosis I, trisomies consisting of two different maternal chromosomes and one paternal chromosome are more common. Subsequent trisomic rescue is achieved by the loss of a paternal chromosome, which makes maternal heterodimers more common. Thus, as described in the background of this article, we found that most of the reports of UPD16 are maternal UPD16.

The specific mechanism of paternal UPD on chromosome 16 in this case is not clear. Based on the abovementioned mechanism of UPD formation, we suggest that it may be caused by an error in meiosis II during the formation of the father's sperm, resulting in the formation of sperm with two chromosomes 16 of paternal origin. As a result of meiotic recombination, the two chromosomes appear as alternate regions of heterozygotes and homozygotes, but there are homozygous regions around the centromere, that is, regional isodisomes. This sperm–ovum union forms a zygote with two paternal chromosomes 16, after which the zygote undergoes trisomy rescue, loses one maternal chromosome 16, and finally develops into an embryo carrying a paternal UPD of chromosome 16.

Incomplete trisomic rescue or monosomic rescue can result in chimeric cell lines, some of which have residual chromosomal trisomies or monosomes, leading to pathogenicity. In this case, we ruled out trisomic or monosomic chimerism by two karyotypic analyses with prenatal amniocentesis and peripheral blood taken from the newborn. At present, only UPD on chromosomes 6, 7, 11, 14, 15, and 20 clearly causes clinical symptoms, and no clearly pathogenic imprinted genes have been identified on chromosome 16. Related cases have reported that chromosome 16 contains 2 clear maternally imprinted genes: *ZNF597* and *NAA60* (17, 18), and genes with possible imprinting effects include *SALL, C16orf57/USB1, ACD*, and *FOXF1*; therefore, paternally derived chromosome 16 uniparental diploidy may be pathogenic (19–22). None of the above six genes was found to be clearly related to the occurrence of diseases. This case also proves that there is no pathogenic maternally imprinted gene on chromosome 16, and WES analysis showed no meaningful gene mutations, which is consistent with the currently observed phenotype. Of course, this conclusion will need to be supported by additional clinical evidence.

Most cases of UPD (16) are of maternal origin, and the available reports of paternal UPD (16) are all complete isodimers. Our report suggests that regional isodimers merging with regional heterodimers can also exist. In the process of sperm formation, errors may also occur during the meiotic phase. The follow-up of this case, with a normal neonatal phenotype, demonstrates the absence of maternally imprinted pathogenic genes on chromosome 16, at least not maternally imprinted pathogenic genes that affect intrauterine fetal development or cause early infant morbidity. In addition, compared with complete isodysomy, homozygous regions of isodysomy combined with heterodysomy are relatively less likely to result in recessive genetic diseases. Clinically, in cases of prenatal diagnosis or postnatal detection of paternal UPD16, the pathogenicity of the UPD itself may not be prioritized, but the fetus/affected child should be recommended for WES analysis to look for genetic mutations. This case may provide some guidance for eugenics on the male side.

## Data Availability Statement

The raw data supporting the conclusions of this article will be made available by the authors, without undue reservation.

## Ethics Statement

The studies involving human participants were approved by the Ethics Committee of the Second Affiliated Hospital of Chongqing Medical University (Approval number: 2021(651)-1.0). Written informed consent was obtained from the minor(s)' legal guardian/next of kin for the publication of any potentially identifiable images or data included in this article.

## Author Contributions

XP carried out study design. YL and LL performed the experiments. XZ wrote the paper. All authors read and approved the final manuscript.

## Conflict of Interest

The authors declare that the research was conducted in the absence of any commercial or financial relationships that could be construed as a potential conflict of interest.

## Publisher's Note

All claims expressed in this article are solely those of the authors and do not necessarily represent those of their affiliated organizations, or those of the publisher, the editors and the reviewers. Any product that may be evaluated in this article, or claim that may be made by its manufacturer, is not guaranteed or endorsed by the publisher.
